# Case report of peritoneal dialysis-associated peritonitis caused by *Mycobacterium abscessus* in a child, successfully treated with dual β-lactam combination therapy

**DOI:** 10.1128/asmcr.00056-25

**Published:** 2025-07-16

**Authors:** Debbie-Ann Shirley, Paola Maurtua-Neumann, Lawrence Shoemaker, Kathryn DeSear, Khalid M. Dousa

**Affiliations:** 1 Division of Infectious Diseases, Department of Pediatrics, University of Florida College of Medicine 50546, Gainesville, Florida, USA; 2 Division of Nephrology, Department of Pediatrics, University of Florida College of Medicine 50546, Gainesville, Florida, USA; 3 Department of Pharmacy, University of Florida Health Shands Hospital 21366, Gainesville, Florida, USA; 4 Division of Infectious Diseases and HIV Medicine, University Hospitals Cleveland Medical Center, Case Western Reserve University 198553, Cleveland, Ohio, USA; Vanderbilt University Medical Center, Nashville, Tennessee, USA

**Keywords:** peritonitis, dual beta-lactam therapy, *Mycobacterium abscessus*

## Abstract

**Background:**

Peritoneal dialysis-associated peritonitis caused by *Mycobacterium abscessus* is a rare and difficult-to-treat infection that frequently results in peritoneal dialysis failure. Since *M. abscessus* is intrinsically resistant to many antibiotics, therapeutic decisions are challenging. While data are limited, a prolonged course of antibiotics with at least two or three agents is recommended, guided by susceptibility testing. Many regimens use amikacin, which can worsen renal function and cause deafness. There is limited safety and efficacy data on newer antimycobacterial medications in children. Safe and well-tolerated options are needed for the treatment of infections caused by *M. abscessus*.

**Case Summary:**

Herein, we present the case of a toddler with dysplastic congenital renal disease, who developed peritoneal dialysis catheter-related *M. abscessus* peritonitis and was treated with a multidrug regimen including two β-lactam antibiotics. This resulted in clinical and microbiologic cure, despite peritoneal failure and transition to hemodialysis. Based on predicted synergy testing, the patient was treated with a combination that included meropenem and ceftaroline for the initial intensified intravenous phase, followed by a regimen that included amoxicillin combined with cefdinir for step-down therapy. This allowed the option of enteral therapy and limited the use of more toxic medications or agents with inadequate information for use in children.

**Conclusion:**

This case highlights the potential benefit of dual β-lactam therapy for the treatment of *M. abscessus* infection as a well-tolerated regimen for a difficult-to-treat infection. To our knowledge, this is the first report using this approach for the treatment of *M. abscessus* peritonitis.

## INTRODUCTION

Nontuberculous mycobacteria (NTM) peritoneal dialysis (PD)-associated peritonitis is a rare and difficult-to-treat infection (1, 2). Complete cure is low, and mortality can be high (3, 4). Less than 20% of patients are able to resume PD following NTM infection (1). Due to biofilm formation, source control with removal of the infected catheter is recommended (1, 2). Multidrug regimens are used to prevent the emergence of resistance. A prolonged multidrug course is often recommended (5); however, drug toxicity can lead to intolerance or poor adherence, and the optimal combinations and duration of therapy have not been established (6). Consultation with an expert in infectious diseases is recommended to guide treatment for NTM infections (1, 2).

NTM peritonitis with *Mycobacterium abscessus* can be particularly challenging to treat due to additional antimicrobial resistance concerns and limited therapeutic options. While no specific guidelines are established for the treatment of peritonitis, society guidelines for the treatment of NTM pulmonary disease classify *M. abscessus* infection based on macrolide susceptibility and suggest a multidrug regimen that includes three or more active drugs guided by *in vitro* testing in the initial phase of treatment (5). There are three genomically characterized subspecies, of which *M. abscessus* subsp. *abscessus* can be particularly challenging to treat due to intrinsic and acquired resistance (1, 2). Alarmingly, treatment outcomes for drug-susceptible *M. abscessus* subsp. *abscessus* are often worse than those for multidrug-resistant tuberculosis (MDR-TB) and may approximate those of extensively drug-resistant tuberculosis (XDR-TB) (7). Elevated minimum inhibitory concentrations to backbone β-lactam treatments (imipenem-cilastatin, cefoxitin) in *M. abscessus* subsp. *abscessus* make achieving pharmacodynamic targets difficult, and the optimal combination regimens and durations are not established (8). While most *M. abscessus* organisms are susceptible to amikacin, the toxicity profile of aminoglycosides limits their use over extended periods (9, 10). Hence, safe and effective treatment options are needed (6). Dual β-lactam therapy is emerging as a promising, well-tolerated synergistic combination for *M. abscessus* infection to restore susceptibility to β-lactam agents (11). We present the case of a child with *M. abscessus* subsp. *abscessus* PD-associated peritonitis successfully treated with dual β-lactam combination therapy.

## CASE PRESENTATION

A 2-year-old toddler with end-stage renal disease secondary to congenital renal dysplasia, managed with nighttime continuous cycler peritoneal dialysis (PD), was admitted to the hospital for treatment of *Pseudomonas aeruginosa* catheter-related exit-site infection, after presenting with one week of thick green mucous discharge at the catheter site. On arrival to the emergency room, vital signs were within normal range, and the patient was well-appearing despite thick purulent discharge emerging from the PD exit site. The abdomen was soft and non-tender to palpation. Initial laboratory findings revealed a white cell count of 13.8 × 10^9^/L (reference range 6.0–17.5 × 10^9^/L), hemoglobin of 9.5 g/dL (reference range 10.5–13.5 g/dL), platelet count of 595 × 10^9^/L (reference range 150–450 × 10^9^/L), and C-reactive protein of 5.48 mg/L (reference range 0–5.00 mg/L). Analysis of the peritoneal fluid from admission did not reveal evidence of peritonitis by cell count (27 total nucleated cells/µL, 18% neutrophils) or Gram stain, and PD fluid culture was negative. Empiric therapy was initiated with intravenous ceftazidime 50 mg/kg (adjusted for renal impairment), intraperitoneal cefepime 125 mg/L, and topical gentamicin 0.1% three times daily over the exit site.

On the night of admission, she developed hypotension and leukocytosis of 22.4 × 10^9^/L (reference range 6.0–17.5 × 10^9^/L) with left shift (69% neutrophils, 18% bands) and an increase of the C-reactive protein to 175 mg/L (reference range 0–5.00 mg/L). Antibiotic therapy was broadened to meropenem, initially dosed at 20 mg/kg (adjusted for renal impairment). Blood culture was negative, leukocytosis improved, and blood pressure stabilized, but she developed a fever of 39.4°C on the fifth day. Evaluation for a new source of fever indicated development of peritonitis with an increase in peritoneal fluid cell count (11,625 total nuclear cells/µL, 77% neutrophils) on day 8 of admission. Enteral linezolid 10 mg/kg every eight hours, intraperitoneal vancomycin 25 mg/L, and intraperitoneal fluconazole 40 mg/L were added to the antibiotic regimen.

**Fig 1:**
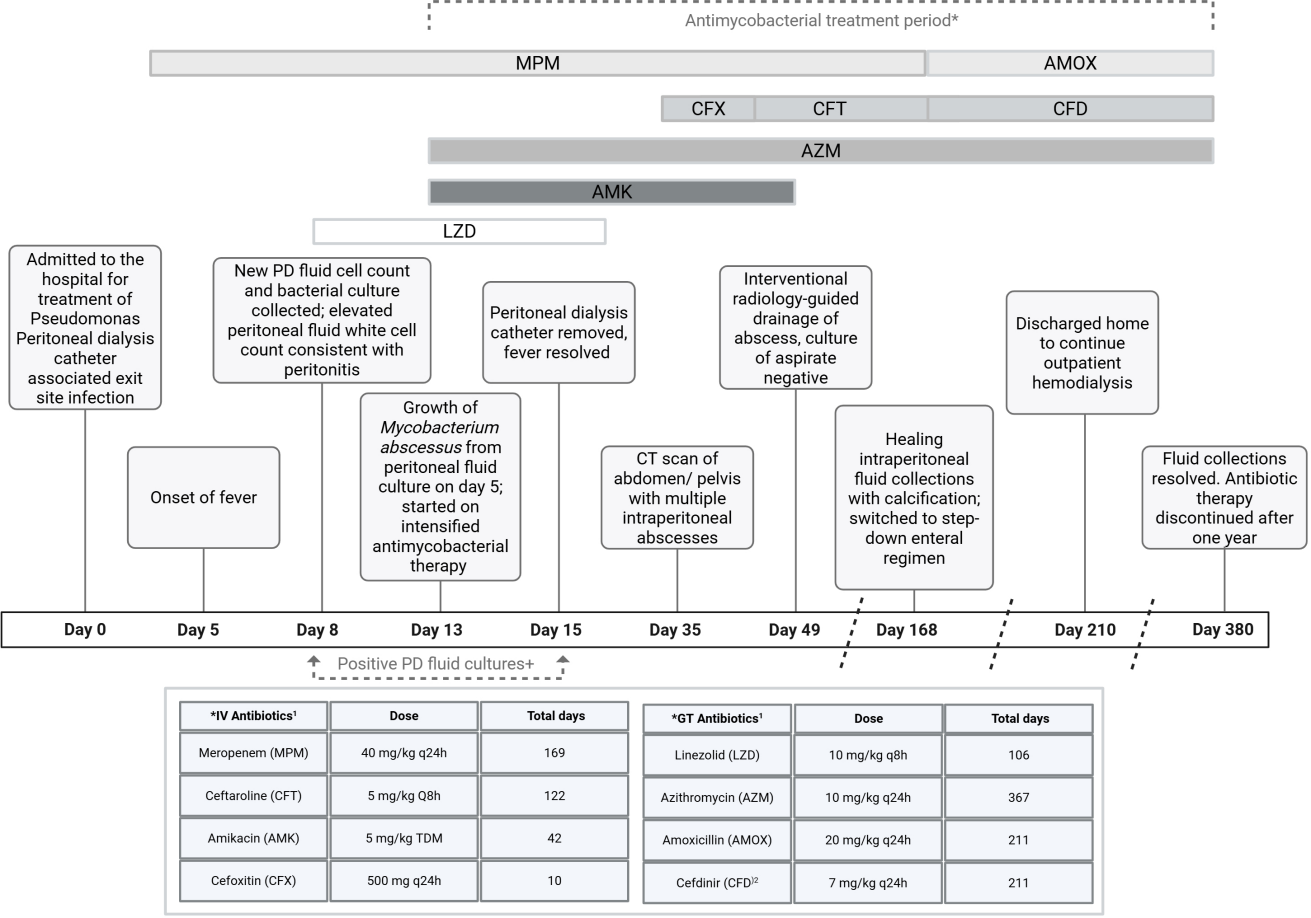
Antimycobacterial treatment timeline. Timeline is not scaled. ^1^Antibiotics administered after hemodialysis. ^2^Cefdinir administered with amoxicillin to optimize synergy. GT, gastric tube; h, hours; IV, intravenous; kg, kilograms; mg, milligrams; PD, peritoneal dialysis; q, every; TDM, therapeutic drug monitoring with redosing based on trough levels. ^+^*Mycobacterium abscessus* grew from all bacterial cultures collected from the peritoneal fluid on day 8, 9, 10, 12, 13, and 14 of admission. The peritoneal dialysis catheter was removed on day 15 of admission.

Repeat bacterial cultures collected from the peritoneal fluid on day 8 of hospitalization detected 1 + acid-fast bacilli five days later, on day 13 of hospitalization. In the setting of a prior negative routine screening for interferon gamma release assay and lack of tuberculosis risk factors, treatment was empirically initiated for rapidly growing nontuberculous mycobacterium (NTM), with the addition of intravenous amikacin 5 mg/kg (re-dosed by drug levels) and azithromycin 10 mg/kg every 24 hours (Fig. 1). The PD catheter was removed on day 15, and the patient was transitioned to thrice weekly hemodialysis. *Mycobacterium abscessus* subsp. *abscessus* was identified via HAIN line-probe assay and isolated from multiple peritoneal fluid cultures prior to catheter removal (Fig. 1). All blood cultures, including bacterial and mycolytic cultures, were negative. The fever resolved following PD catheter removal, and inflammatory markers improved. CT scan of the abdomen and pelvis demonstrated multiple intraperitoneal fluid collections concerning for abscess formation, with the most well-defined collection located in the right anterior hemipelvis measuring 3.4 × 1.9 × 3.2 cm. The pediatric surgical team was consulted, but no surgical intervention was initially advised to limit the risk of creating draining fistulas.

Following the results of susceptibility testing, therapy was optimized to meropenem 40 mg/kg every 24 hours (adjusted for renal impairment), cefoxitin 500 mg/kg every 24 hours (adjusted for renal impairment), azithromycin 10 mg/kg every 24 hours, and amikacin 5 mg/kg (re-dosed by drug levels) on day 36, utilizing dual β-lactam therapy to optimize synergy between cefoxitin and meropenem (Table 1). Additional dual β-lactam synergy testing further suggested enhanced synergy with the combination of imipenem or meropenem and ceftaroline (Table 2); hence, cefoxitin was switched to intravenous ceftaroline 5 mg/kg every 24 hours (adjusted for renal impairment) on day 47. The largest fluid collection was ultimately drained by interventional radiology on day 49, and the fluid culture was sterile. Amikacin was discontinued after six weeks of therapy on day 55, due to decreasing otoacoustic emissions.

**TABLE 1:** *Mycobacterium abscessus* subsp. *abscessus* susceptibility testing*^a^*

Antibiotic	Minimal inhibitory concn (µg/mL)	Interpretation*^b^*
Amikacin	8	Susceptible
Cefoxitin	32	Intermediate
Ciprofloxacin	4	Resistant
Clarithromycin	0.25	Susceptible
Clofazimine	0.25	No CLSI interpretation available
Doxycycline	>8	Resistant
Imipenem	16	Intermediate
Linezolid	16	Intermediate
Moxifloxacin	>4	Resistant
Tigecycline	0.25	No CLSI interpretation available
Trimethoprim/sulfamethoxazole	>4	Resistant

^*a*^ Macrolide screen: macrolide susceptibility is predicted, no detectable mutation in *rrl* gene. Aminoglycoside screen: aminoglycoside susceptibility is predicted, no detectable mutation in *rrs* gene. Inducible macrolide resistance: inducible macrolide resistance is not predicted; a cytosine has been detected at position 28 (C28) of the *erm*(41) gene.

^*b*^ Breakpoints from the CLSI M24S, 2nd ed. (12). Organism identification performed via HAIN line-probe assay. Macrolide and aminoglycoside resistance screens were performed using the GenoType NTM-DR molecular assay. Performed at the University of Florida Health Pathology Laboratories Nontuberculosis Mycobacterial Lab.

**TABLE 2:** *Mycobacterium abscessus* subsp. *abscessus* dual-β lactam synergy testing*^a^*

Antibiotic (µg/mL)	Test method	Minimal inhibitory concn (µg/mL)
Imipenem	Alone	16
Cefuroxime	Alone	256
Cefdinir	Alone	128
Ceftaroline	Alone	64
Imipenem + cefuroxime	Cefuroxime fixed at 4	0.5
Imipenem + cefdinir	Cefdinir fixed at 4	0.5
Imipenem + ceftaroline	Ceftaroline fixed at 4	0.25
Imipenem + amoxicillin + relebactam	Amoxicillin fixed at 8, relebactam at 4	≤0.25
Cefdinir + amoxicillin	Amoxicillin fixed at 8	1

^*a*^ MICs of ceftaroline, imipenem, cefuroxime, and cefdinir were determined using broth microdilution. Approximately 5 × 10^5^ CFU/ml were inoculated into Middlebrook 7H9 broth supplemented with 10% (vol/vol) oleic albumin dextrose catalase and 0.05% (vol/vol) Tween 80. When more than two drugs were combined, the second drug was added at a fixed concentration of 4 μg/mL or 8 μg/mL to serial dilutions of imipenem, meropenem, or cefdinir. Isolates were incubated with test agents at 30°C for 3 to 7 days, and MIC was defined as the lowest antibiotic concentration that prevented visible bacterial growth. Active moiety compounds, and not pro-drug, e.g., ceftaroline (unphosphorylated) and not ceftaroline fosamil, were used. Performed at Case Western Reserve University at the Cleveland VA Medical Center.

Abdominal imaging findings continued to improve on combination therapy, showing healing of the fluid collections and peritoneal inflammation with calcification. Inflammatory markers declined further, and C-reactive protein normalized by day 167 of admission. Based on the expanded synergy testing (Table 2), the patient was transitioned to an all-enteral step-down regimen with azithromycin 10 mg/kg every 24 hours, amoxicillin 20 mg/kg every 24 hours (adjusted for renal impairment), and cefdinir 7 mg/kg every 24 hours (adjusted for renal impairment). The patient tolerated the therapy well with good adherence. Following prolonged hospitalization, the patient was discharged home after 210 days. Additional complications that arose during the hospital course included ventricular fibrillation from hyperkalemia requiring defibrillation and the development of pulmonary hypertension requiring oxygen supplementation and pulmonary vasodilator therapy. The patient completed a total of 12 months of antimycobacterial therapy from the day of catheter removal. Repeat ultrasound at the end of therapy and two months after showed resolution of all fluid collections.

## DISCUSSION

In this report, we describe the use of dual β-lactam synergistic combination therapy as part of a multidrug regimen to successfully treat a child with *Mycobacterium abscessus* subsp. *abscessus* PD-associated peritonitis.

*M. abscessus* is considered among the most pathogenic and drug-resistant of the rapidly growing NTM (13). In our case, evidence of inducible or mutational macrolide resistance was not shown; hence, we were fortunately able to treat with a macrolide-containing regimen for the entire course. Most *M. abscessus* isolates are also susceptible to amikacin, but development of side effects, such as otovestibular toxicity, often precludes prolonged use, as noted in our case. Susceptibility to all other tested antibiotics was reduced (Table 1); hence, we sought a novel option that could be safely administered to children over an extended period.

Our therapeutic rationale for using combination synergistic dual β-lactam therapy with ceftaroline and meropenem during the initial intensified treatment phase is derived from accumulating clinical reports and encouraging observations using this approach (11, 14–17). This includes a single pediatric report of a 3-year-old child with bronchiectasis and *M. abscessus* pulmonary infection treated with a multidrug regimen that contained ceftaroline to enhance the activity of meropenem, resulting in both clinical and microbiologic cure (14). *M. abscessus* is known to produce β-lactamases that lead to variable β-lactam susceptibility (18). *In vitro* studies demonstrate that imipenem combined with ceftaroline significantly lowers the imipenem minimum inhibitory concentration of clinical isolates. Both drugs target the same peptidoglycan synthesis enzymes. A plausible mechanism for synergy may be explained by either binding of multiple targets with differential inhibition of enzymes involved in peptidoglycan synthesis or blocking of β-lactam hydrolysis to restore susceptibility (11, 19–21). Though not tested in our case, meropenem was preferentially used given its lower risk of inducing seizures in children (22). Our decision to use amoxicillin with cefdinir for step-down enteral therapy, after infection was deemed well-controlled, was based on *in vitro* testing (Table 2) and reports showing synergy with amoxicillin and other β-lactam combinations, such as imipenem (17, 23). The synergy testing uses varying concentrations in doubling dilutions of one β-lactam with a fixed dose of another β-lactam agent. The minimal inhibitory concentration (MIC) of monotherapy is compared to the MIC in the presence of a fixed-dose second agent to determine whether there is an appreciable drop in MIC. This was the case for both combination regimens utilized, where the imipenem MIC dropped from 16 mcg/mL when tested alone to an MIC of 0.5 mcg/mL when tested with a fixed concentration of 4 mcg/mL of cefdinir. The cephem concentration was fixed at 4 μg/mL because this is the level where synergy is observed (19, 20). Similarly, the cefdinir concentration dropped from 128 mcg/mL to 1 mcg/mL when tested with a fixed concentration of 8 mcg/mL amoxicillin.

Antimycobacterial treatment dosages for children are not well established. When available, guideline-based (24) pediatric dosages were renally adjusted and increased for growth throughout the treatment course. There is a paucity of data on the pharmacokinetic profiles of these medications in patients receiving renal replacement therapies. Aggressive dosing using maximum tolerable doses should be considered when using oral β-lactams for synergy, while carefully considering dose-limiting toxicities that can occur with many of the β-lactams (25, 26).

The optimal duration of therapy is also unknown, but often exceeds one year (5, 24). We opted to treat for this duration to decrease the risk of relapse of infection and increase the chance of a successful outcome as a renal transplant candidate. Alternative approaches were considered. Newer antimycobacterial agents include the tetracycline derivatives eravacycline and omadacycline, though safety is not established in children. Similarly, there is limited data on using the diarylquinoline antibiotic bedaquiline, which is associated with cardiotoxicity and is difficult to access. The riminophenazine antibiotic, clofazimine, and bacteriophage therapy are investigational therapies confined to expanded access pathways. In contrast, β-lactam antibiotics are widely available and are among the best tolerated antibiotics with an excellent established profile.

In summary, we report the successful treatment of *M. abscessus* subsp. *abscessus* PD-associated peritonitis complicated by multiple intra-abdominal abscesses using dual β-lactam synergistic therapy as part of a multidrug approach. A similar strategy has been used to treat other challenging cases of *M. abscessus* infection. To our knowledge, this is the first report using dual β-lactam therapy to treat peritonitis and provides further support for use in hard-to-treat infections based on the safety of this approach. Larger studies and clinical investigations are needed to determine the best combinations, dosages, and durations of treatment for this promising antimycobacterial synergism.

## References

[1] Li PT, Chow KM, Cho Y, Fan S, Figueiredo AE, Harris T, Kanjanabuch T, Kim YL, Madero M, Malyszko J, Mehrotra R, Okpechi IG, Perl J, Piraino B, Runnegar N, Teitelbaum I, Wong JW, Yu X, Johnson DW. 2022. ISPD peritonitis guideline recommendations: 2022 update on prevention and treatment. Perit Dial Int 42:110–153. doi:10.1177/08968608221080586 35264029

[2] Warady BA, Same R, Borzych-Duzalka D, Neu AM, El Mikati I, Mustafa RA, Begin B, Nourse P, Bakkaloglu SA, Chadha V, Cano F, Yap HK, Shen Q, Newland J, Verrina E, Wirtz AL, Smith V, Schaefer F. 2024. Clinical practice guideline for the prevention and management of peritoneal dialysis associated infections in children: 2024 update. Perit Dial Int 44:303–364. doi:10.1177/08968608241274096 39313225

[3] Hashimoto N, Kani N, Makino S, Naka T, Miyakawa H, Okamoto K, Uwatoko R, Bessho S, Iio R, Ueda Y, Hayashi T. 2023. Fatal peritoneal dialysis-associated peritonitis caused by Mycobacterium mageritense: a case report with review. Ren Replace Ther 9:5. doi:10.1186/s41100-023-00457-4

[4] Shu CC, Wang JT, Wang JY, Yu CJ, Lee LN. 2012. Mycobacterial peritonitis: difference between non-tuberculous mycobacteria and Mycobacterium tuberculosis. Clin Microbiol Infect 18:246–252. doi:10.1111/j.1469-0691.2011.03547.x 21631640

[5] Daley CL, Iaccarino JM, Lange C, Cambau E, Wallace RJ, Andrejak C, Böttger EC, Brozek J, Griffith DE, Guglielmetti L, Huitt GA, Knight SL, Leitman P, Marras TK, Olivier KN, Santin M, Stout JE, Tortoli E, van Ingen J, Wagner D, Winthrop KL. 2020. Treatment of nontuberculous mycobacterial pulmonary disease: an official ATS/ERS/ESCMID/IDSA clinical practice guideline. Eur Respir J 56:2000535. doi:10.1183/13993003.00535-2020 32636299 PMC8375621

[6] Adjemian J, Prevots DR, Gallagher J, Heap K, Gupta R, Griffith D. 2014. Lack of adherence to evidence-based treatment guidelines for nontuberculous mycobacterial lung disease. Ann Am Thorac Soc 11:9–16. doi:10.1513/AnnalsATS.201304-085OC 24236749 PMC3972983

[7] Pasipanodya JG, Ogbonna D, Ferro BE, Magombedze G, Srivastava S, Deshpande D, Gumbo T. 2017. Systematic review and meta-analyses of the effect of chemotherapy on pulmonary Mycobacterium abscessus outcomes and disease recurrence. Antimicrob Agents Chemother 61:e01206-17. doi:10.1128/AAC.01206-17 28807911 PMC5655093

[8] le Run E, Tettelin H, Holland SM, Zelazny AM. 2024. Evolution towards extremely high β-lactam resistance in Mycobacterium abscessus outbreak strains. bioRxiv:2024.05.08.593223. doi:10.1101/2024.05.08.593223 PMC11459939 39254295

[9] Aznar ML, Marras TK, Elshal AS, Mehrabi M, Brode SK. 2019. Safety and effectiveness of low-dose amikacin in nontuberculous mycobacterial pulmonary disease treated in Toronto, Canada. BMC Pharmacol Toxicol 20:37. doi:10.1186/s40360-019-0302-1 31159865 PMC6547538

[10] Modongo C, Pasipanodya JG, Zetola NM, Williams SM, Sirugo G, Gumbo T. 2015. Amikacin concentrations predictive of ototoxicity in multidrug-resistant tuberculosis patients. Antimicrob Agents Chemother 59:6337–6343. doi:10.1128/AAC.01050-15 26248372 PMC4576092

[11] Nguyen DC, Dousa KM, Kurz SG, Brown ST, Drusano G, Holland SM, Kreiswirth BN, Boom WH, Daley CL, Bonomo RA. 2021. “One-two punch”: synergistic ß-Lactam combinations for Mycobacterium abscessus and target redundancy in the inhibition of peptidoglycan synthesis enzymes. Clin Infect Dis 73:1532–1536. doi:10.1093/cid/ciab535 34113990 PMC8677594

[12] Anonymous. 2023. Performance standards for susceptibility testing of Mycobacteria, Nocardia spp., and other aerobic actinomycetes. 2nd Edition. CLSI supplement M24S. Clinical and Laboratory Standards Institute. 31339680

[13] Johansen MD, Herrmann JL, Kremer L. 2020. Non-tuberculous mycobacteria and the rise of Mycobacterium abscessus. Nat Rev Microbiol 18:392–407. doi:10.1038/s41579-020-0331-1 32086501

[14] Becken B, Dousa KM, Johnson JL, Holland SM, Bonomo RA. 2024. Dual β-lactam for treatment of pulmonary Mycobacterium abscessus in a child. Antimicrob Agents Chemother 68:e0031924. doi:10.1128/aac.00319-24 38757973 PMC11232406

[15] Alahmdi B, Dousa KM, Kurz SG, Kaufman A, Bonomo RA, Taimur S. 2023. Eradicating pulmonary Mycobacterium abscessus: the promise of dual β-Lactam therapy. Open Forum Infect Dis 10:ofad312. doi:10.1093/ofid/ofad312 37383246 PMC10296056

[16] Griffith DE, Daley CL. 2022. Treatment of Mycobacterium abscessus pulmonary disease. CHEST 161:64–75. doi:10.1016/j.chest.2021.07.035 34314673

[17] Moguillansky N, DeSear K, Dousa KM. 2023. A 40-year-old female with Mycobacterium abscessus successfully treated with a dual beta-lactam combination. Cureus 15:e40993. doi:10.7759/cureus.40993 37503487 PMC10371195

[18] Story-Roller E, Maggioncalda EC, Cohen KA, Lamichhane G. 2018. Mycobacterium abscessus and β-Lactams: emerging insights and potential opportunities. Front Microbiol 9:2273. doi:10.3389/fmicb.2018.02273 30319581 PMC6167491

[19] Dousa KM, Kurz SG, Taracila MA, Bonfield T, Bethel CR, Barnes MD, Selvaraju S, Abdelhamed AM, Kreiswirth BN, Boom WH, Kasperbauer SH, Daley CL, Bonomo RA. 2020. Insights into the l,d-transpeptidases and d,d-carboxypeptidase of Mycobacterium abscessus: ceftaroline, imipenem, and novel diazabicyclooctane inhibitors. Antimicrob Agents Chemother 64:e00098-20. doi:10.1128/AAC.00098-20 32393499 PMC7526840

[20] Dousa KM, Nguyen DC, Kurz SG, Taracila MA, Bethel CR, Schinabeck W, Kreiswirth BN, Brown ST, Boom WH, Hotchkiss RS, Remy KE, Jacono FJ, Daley CL, Holland SM, Miller AA, Bonomo RA. 2022. Inhibiting Mycobacterium abscessus cell wall synthesis: using a novel diazabicyclooctane β-lactamase inhibitor to augment β-lactam action. MBio 13:e0352921. doi:10.1128/mbio.03529-21 35073757 PMC8787486

[21] Pandey R, Chen L, Manca C, Jenkins S, Glaser L, Vinnard C, Stone G, Lee J, Mathema B, Nuermberger EL, Bonomo RA, Kreiswirth BN. 2019. Dual β-lactam combinations highly active against Mycobacterium abscessus complex in vitro. MBio 10:e02895-18. doi:10.1128/mBio.02895-18 30755518 PMC6372805

[22] Cannon JP, Lee TA, Clark NM, Setlak P, Grim SA. 2014. The risk of seizures among the carbapenems: a meta-analysis. J Antimicrob Chemother 69:2043–2055. doi:10.1093/jac/dku111 24744302

[23] Lopeman RC, Harrison J, Rathbone DL, Desai M, Lambert PA, Cox JAG. 2020. Effect of amoxicillin in combination with imipenem-relebactam against Mycobacterium abscessus. Sci Rep 10:928. doi:10.1038/s41598-020-57844-8 31988293 PMC6985242

[24] Haworth CS, Banks J, Capstick T, Fisher AJ, Gorsuch T, Laurenson IF, Leitch A, Loebinger MR, Milburn HJ, Nightingale M, Ormerod P, Shingadia D, Smith D, Whitehead N, Wilson R, Floto RA. 2017. British thoracic society guidelines for the management of non-tuberculous mycobacterial pulmonary disease. Thorax 72:ii1–ii64. doi:10.1136/thoraxjnl-2017-210927 29054853

[25] Fonseca W, Hoppu K, Rey LC, Amaral J, Qazi S. 2003. Comparing pharmacokinetics of amoxicillin given twice or three times per day to children older than 3 months with pneumonia. Antimicrob Agents Chemother 47:997–1001. doi:10.1128/AAC.47.3.997-1001.2003 12604533 PMC149282

[26] Bowlware KL, McCracken GH, Lozano-Hernandez J, Ghaffar F. 2006. Cefdinir pharmacokinetics and tolerability in children receiving 25 mg/kg once daily. Pediatr Infect Dis J 25:208–210. doi:10.1097/01.inf.0000202210.22512.88 16511381

